# The Therapeutic Effects of the Chinese Herbal Medicine, Lang Chuang Fang Granule, on Lupus-Prone MRL/*lpr* Mice

**DOI:** 10.1155/2016/8562528

**Published:** 2016-02-29

**Authors:** Kai-Peng Huang, Zhi-Hao Zhang, Rui-Ming Li, Xiao Chen

**Affiliations:** Department of Pharmacy, The First Affiliated Hospital, Sun Yat-sen University, Guangzhou 510080, China

## Abstract

Systemic lupus erythematosus (SLE) is a chronic autoimmune disease that leads to severe multiorgan damage. Lang Chuang Fang (LCF) is a Chinese herbal medicine that is clinically prescribed for treating SLE. In this study, we examined the therapeutic effects of LCF granule on lupus-prone MRL/*lpr* mice. Female mice were randomly separated into six groups, and LCF treatment groups received LCF granule at the dosage of 0.97 g/kg/d, 1.95 g/kg/d, and 3.90 g/kg/d, respectively. Here, we found that, compared to the MRL/*lpr* mice, both the spleen coefficient and thymus coefficient were reduced in the LCF granule-treated mice. There was a marked downregulation in CRP and anti-dsDNA autoantibody and an evident upregulation of CH50 in LCF granule-treated mice. LCF granule treatment also obviously reduced the proteinuria, BUN, and SCr levels in MRL/*lpr* mice at the dosage of 0.97 g/kg/d, 1.95 g/kg/d, and 3.90 g/kg/d, indicating that LCF granule alleviated the renal injury of MRL/*lpr* mice. Furthermore, LCF granule decreased p65 NF-*κ*B levels and increased Sirt1 and Nrf2 levels in the kidney tissues of MRL/*lpr* mice, which might elucidate the beneficial effects of LCF on lupus nephritis. In conclusion, this study demonstrates that LCF granule has therapeutic effects on lupus-prone MRL/*lpr* mice.

## 1. Introduction

Systemic lupus erythematosus (SLE) is a chronic autoimmune disease characterized by massive autoantibodies production, immune-complex deposition in micrangium, and inflammatory cells infiltration that lead to severe multiorgan damage [1]. The morbidity of SLE has significant difference in age, sex, race, and areas [1–3]. SLE is much more common in women than in men, with a female-to-male ratio of about 9 : 1 as commonly reported [1–3]. With the progress in medical care, there has been a striking rise in life quality for SLE in recent years with a mean estimated 5-year survival of 82%–90% [4].

The pathogenesis of SLE is still not clearly elucidated, and immunity, hereditary, environment, and sex hormone were thought to be the probable inducers [5]. Among these, the dysfunction of the immune system is crucial for SLE [6]. There are multiple immunoregulatory abnormalities, high titer of autoantibodies, and systemic inflammatory response in patients with SLE, including decreased lymphocytes, maladjusted macrophages and natural killer cells, loss of immune tolerance, reduced complements, and the abnormality of lymphokines, such as interleukin-1 (IL-1), IL-2, and tumor necrosis factor-*α* [5, 6].

At present, most patients with SLE need long-term treatment of glucocorticoids, antimalarial drugs, and immunosuppressive agents, such as azathioprine or cyclophosphamide, to control disease activity [7]. Due to the complications of SLE and the severe side effects of the above drugs including secondary infection, Cushing's syndrome, osteoporosis, and diabetes, drug therapy of SLE has clinically become an important issue [7, 8]. Therefore, it is urgent to find safe and effective drugs with fewer side effects. The therapeutic effects of traditional Chinese medicine (mostly by taking Chinese herbal medicine) on SLE have gained full affirmation of researchers both at home and abroad [9–14]. In clinical application, Chinese herbal medicine has been prescribed as a combination of more than two herbal drugs to obtain any additive effects and to diminish the possible adverse responses [9–14].

According to the traditional Chinese medicine, SLE can be defined as rheumatism, edema, and palpitation of heart due to different organic damage and in general divided into excessive syndromes and deficient syndromes [12]. The traditional Chinese prescription Lang Chuang Fang (LCF) is originated from the clinical experience of Professor Ye, a well-known specialist in nephrosis in China. Based on the long-term investigations on the pathogenesis and treatment of chronic kidney disease, Professor Ye invented LCF according to the therapeutic principle of “heat-clearing and detoxifying, promoting blood circulation and removing blood stasis.” LCF is composed of seven important herbals including* Hedyotis diffusa *Willd.,* Arnebia euchroma *(Royle) Johnst.,* Scutellaria barbata *D. Don,* Rehmannia glutinosa *Libosch, and* Salvia miltiorrhiza* Bge. [15]. Hospital preparation LCF granule has clinically been applied in the First Affiliated Hospital, Sun Yat-sen University, for treating SLE for more than 30 years, which showed evident curative effects with lower recurrence and fewer side effects [15].

Professor Ye and his research team also performed series of experiments on lupus-like mice to elucidate the precise mechanisms of LCF [16–18]. They found that LCF could inhibit the immune system through decreasing the expression of CD134 in T lymphocytes and CD134L in B lymphocytes, interfering with the signaling molecules, such as CD4, CD8, CD19, CD23, and CD40, and downregulating RANTES levels to prevent the activation of lymphocytes and reduce autoantibodies formation, which eventually relieve the pathological progression of SLE [16, 17]. Additionally, LCF also inhibited the lymphocyte subsets in the spleen of lupus-like BXSB mice to improve the dysfunction and pathological changes of kidney and lung [18]. To further clarify the mechanism of LCF, the current study was undertaken to observe the therapeutic effects of LCF on lupus-prone MRL/*lpr* mice. We found that LCF granule reduced the spleen and thymus coefficients, decreased C-reactive protein (CRP) and anti-dsDNA autoantibody levels, and alleviated renal impairment of MRL/*lpr* mice.

## 2. Materials and Methods

### 2.1. MRL/*lpr* Mice

Female lupus-prone MRL/*lpr* mice aged eight weeks were provided by Guangdong Medical Laboratory Animal Center (Guangzhou, China). The experiments were carried out in the Experimental Animal Center of Sun Yat-sen University (certification number: SYXK-2012-0081; Guangzhou, China) and approved by the Ethics Committee on the Care and Use of Laboratory Animals of Sun Yat-sen University (Guangzhou, China).

### 2.2. Reagents and Antibodies

Urine protein quantitation kit (Lot: 20150407), blood urea nitrogen (BUN) determination kit (Lot: 20150401), and serum creatinine (SCr) determination kit (Lot: 20150402) were purchased from Nanjing Jiancheng Bioengineering Institute (Nanjing, China). ELISA kit for mouse anti-dsDNA autoantibody (Lot: 1503251), ELISA kit for mouse CH50 (Lot: 1408212), ELISA kit for mouse CRP (Lot: 1408251), and ELISA kit for mouse IgE (Lot: 1408081) were provided by Shanghai Westang Bio-Tech Co., Ltd. (Shanghai, China). Anti-mouse CD3 FITC (Clone: 145-2c11, Lot: E00058-1631), anti-mouse CD8 APC (Clone: 53-6.7, Lot: E07055-1634), and anti-mouse CD54 PE (Clone: YN1/1.7.4, Lot: E01295-1633) were purchased from eBioscience Inc. (San Diego, CA, USA). Antibodies against p65 NF-*κ*B, Sirt1, and Nrf2 were provided by Santa Cruz Biotechnology (Santa Cruz, CA, USA).

### 2.3. Dosage of LCF Granule and Positive Drugs

LCF granule was prepared by the Guangdong Traditional Chinese Medical Science Institute (Lot: 140417). Clinically, LCF granule was administrated orally with the dosage of 5.0 g, three times per day, and the total dosage was 15.0 g per day. According to the dose conversion method in “Chinese medicine pharmacology research methodology” [19], the clinical equivalent dose of LCF granule for mice is 1.95 g/kg/d. And the dosage of LCF granule was grouped as 0.97 g/kg/d, 1.95 g/kg/d, and 3.90 g/kg/d.

The positive drug hydroxychloroquine sulfate tablets were purchased from Shanghai Zhongxi Pharmaceutical Co., Ltd. (Shanghai, China), which was mainly used to treat rheumatoid arthritis, juvenile chronic arthritis, SLE, and skin lesions caused by sunlight. The daily dosage of hydroxychloroquine sulfate tablet is 0.4 g. After dose conversion, the clinical equivalent dose of hydroxychloroquine sulfate for mice is 52.00 mg/kg/d.

The other positive drug Langchuang Pill was purchased from Changchun Haiwai Pharmaceutical Co., Ltd. (Changchun, China), which was mainly used to treat the “stagnation of pyretic toxicity”-induced SLE. The daily dosage of Langchuang Pill is 0.54 g. After dose conversion, the clinical equivalent dose of Langchuang Pill for mice is 1.40 g/kg/d.

At the time of the experiment, the above drugs were prepared in distilled water to the required concentration.

### 2.4. Experimental Protocol

After being fed with regular diet for one week, the MRL/*lpr* mice were randomly divided into lupus model group (MRL/*lpr*), hydroxychloroquine sulfate tablet group (HCQ), Langchuang Pill group (LCP), low dose of LCF granule group (0.97 g/kg/d; LCF Low), medium dose of LCF granule group (1.95 g/kg/d; LCF Medium), and high dose of LCF granule group (3.90 g/kg/d; LCF High). Every group contains 10 mice. Animals received the indicated drugs between 9:00 am and 10:00 am once a day for continuous 4 weeks. The MRL/*lpr *model mice received equivalent distilled water. During the gavage period, mice were continually given free access to water and standard laboratory chow.

After administration on day 27, the mice were housed in metabolic cage to gather the 24-hour urine. At the termination of the experiments, the mice were weighed. All animals were sacrificed after anesthesia, and the blood samples were collected through the orbit. Part of the blood samples was put in the EDTAK2 anticoagulant tube to detect the expression of CD3^+^, CD8^+^, and CD54^+^ on the surface of peripheral blood lymphocytes. The rest were put in ordinary glass tube and then the serum was separated to detect the anti-dsDNA autoantibody, CH50, CRP, IgE, BUN, and SCr levels. Then, the mice were sacrificed by cervical dislocation. Spleen and thymus were excised and weighted to calculate the coefficients. Kidney samples were quickly excised and frozen in liquid nitrogen and then stored at −80°C.

### 2.5. Flow Cytometry

Flow cytometric analysis was performed using FITC-conjugated CD3 anti-mouse antibody, APC-conjugated CD8 anti-mouse antibody, and PE-conjugated CD54 anti-mouse antibody. 100 *μ*L of PBS with the above antibodies diluted to the equivalent of 1 *μ*g/1 × 10^6^ cells was added to the cells. After 30 min, cells were washed with PBS and analyzed by flow cytometry using Cytomics*™* FC 500 (Beckman Coulter; Brea, CA, USA) to calculate the proportion of CD3^+^, CD8^+^, and CD54^+^ in the lymphocytes of the peripheral blood. Data were analyzed using the CellQuest software program (Becton-Dickinson; Franklin Lakes, NJ, USA).

### 2.6. Anti-dsDNA Autoantibody, CRP, CH50, and IgE Detection

Anti-dsDNA autoantibody levels were quantified by ELISA. dsDNA was isolated by S1 nuclease treatment of phenol-extracted calf thymus DNA. 96-well ELISA plates were then coated with 5 *μ*g/mL of calf thymus dsDNA and incubated at 37°C overnight. The plates were washed with PBS containing 0.05% Tween. Serum was added to each well in a 1 : 100 dilution and incubated for 45 min at room temperature. After washing, HRP-conjugated goat anti-mouse IgG was added and incubated for 45 min. After thorough washing, the results were reported as the absorbance at 380 nm at a 1 : 100 serum dilution. Serum levels of CRP, CH50, and IgE in the supernatants were also quantified by ELISA following the manufacturers' instructions.

### 2.7. Evaluation of Renal Injury

Urinary protein, BUN, and SCr levels were determined using commercial kits following the manufacturers' instructions to evaluate the renal function.

### 2.8. Western Blot Assay

Western blot analysis was performed to detect the protein expression of p65 NF-*κ*B, Sirt1, and Nrf2 in the kidney tissues of MRL/*lpr* mice. Briefly, equal amounts of protein samples from kidney cortex fragments were subjected to SDS-PAGE. Immunoreactive bands were visualized using an enhanced chemiluminescence substrate (Thermo Fisher Scientific; Rockford, IL, USA) with a GE ImageQuant LAS 4000 mini (GE healthcare; Waukesha, WI, USA). The intensity of protein bands were quantified using a Gel Doc XR System (Bio-Rad; Hercules, CA, USA).

### 2.9. Statistical Analysis

Values were expressed as means ± SDs. All data were assessed by the Graphpad Prism 5.0 software. Unpaired Student's *t*-test was used for comparison between two groups. For multiple comparisons, data were analyzed by one-way ANOVA with* post hoc* multiple comparisons. Independent experiments were performed at least thrice with similar results. *P* < 0.05 was considered statistically significant.

## 3. Results

### 3.1. LCF Granule Treatment Reduced Spleen and Thymus Coefficients in MRL/*lpr* Mice

Splenomegaly and thymus disease are known manifestations of* fas* deficiency and are considered to be clinical markers of lupus patients and MRL/*lpr* mice [20]. At 13 weeks of age, the mice were weighed and sacrificed, and the spleen and thymus weights were measured. The spleen weight to body weight ratio and thymus weight to body weight ratio were indicated as the lesion degree of spleen and thymus, respectively. As the data in Table 1 showed, compared to the MRL/*lpr* mice, high dose treatment of LCF granule obviously reduced the spleen coefficient and medium dose of LCF granule decreased the thymus coefficient. Additionally, the reducing effect of high dose LCF granule on spleen coefficient was similar to the positive drug Langchuang Pill (Table 1).

### 3.2. The Effects of LCF Granule on CD3^+^, CD8^+^, and CD54^+^ Expression on the Surface of Peripheral Blood Lymphocytes in MRL/*lpr* Mice

CD3 is the symbol of mature T lymphocyte, CD8 is the classification markers of T lymphocyte subsets, and CD54, also known as intercellular cell adhesion molecule-1, is a member of immunoglobulin superfamily that participate in immune reaction and inflammatory reaction [18]. The increase of CD3^+^ indicates the augment of total T lymphocyte amount. And the overexpression of CD8^+^ T lymphocyte, especially the T lymphocyte secreting IL-4, promotes the differentiation and maturation of B lymphocyte and secretion of immunoglobulin, increases the formation of immune-complex, and enhances humoral immune response. Compared to the MRL/*lpr* mice, although the expression of CD3^+^ and CD8^+^ on the surface of peripheral blood lymphocytes showed reducing trend after all three dosages of LCF granule treatment, there was no statistical significance among the results (Table 2). As for CD54^+^, LCF granule had no influence on its expression (Table 2).

### 3.3. Modulation of CRP in MRL/*lpr* Mice following LCF Granule Administration

**Figure 1:**
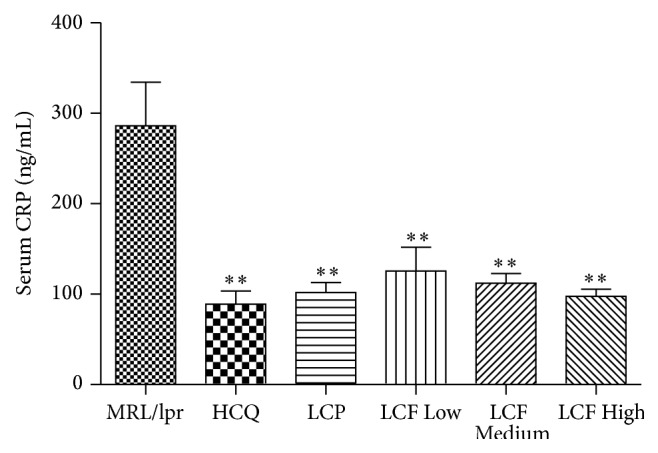
Modulation of CRP in MRL/*lpr* mice following LCF granule administration. Serum levels of CRP were quantified by ELISA following the manufacturers' instructions. MRL/*lpr*: lupus model group; HCQ: hydroxychloroquine sulfate tablet group; LCP: Langchuang Pill group; LCF Low: low dose of LCF granule group (0.97 g/kg/d); LCF Medium: medium dose of LCF granule group (1.95 g/kg/d); LCF High: high dose of LCF granule group (3.90 g/kg/d). Data are expressed as means ± SDs, *n* = 10. ^*∗∗*^ *P* < 0.01 versus MRL/*lpr*.

CRP is a major mediator of several autoimmune and inflammatory diseases. We thus compared its concentration in the serum of MRL/*lpr* mice and MRL/*lpr* mice treated with LCF granule. As expected, LCF granule treatment dose-dependently reduced CRP concentrations in MRL/*lpr* mice (Figure 1). Additionally, the reducing effect of high dose treatment of LCF granule on CRP levels was similar to the positive drug Langchuang Pill (Figure 1).

### 3.4. The Effects of LCF Granule on Anti-dsDNA Autoantibody Production and CH50 Levels

Activation of the immune system by aberrant self-nucleic acid has emerged as another fundamental mechanism in the pathogenesis of SLE [5]. We measured the anti-dsDNA autoantibody, which is the hallmark of SLE and plays important pathogenic roles in lupus nephritis (LN) [5]. LCF granule treatment decreased the concentration of the serum anti-dsDNA autoantibody at the dosage of 0.97 g/kg/d, 1.95 g/kg/d, and 3.90 g/kg/d (Table 3). Additionally, the reducing effect of high dose treatment of LCF granule on anti-dsDNA autoantibody was similar to Langchuang Pill (Table 3). Strikingly, LCF granule treatment also evidently increased the serum CH50 levels with the above three dosages (Table 3), while, for IgE, LCF granule slightly downregulated its levels with no statistical significance (Table 3).

### 3.5. LCF Granule Alleviated the Renal Injury of the Lupus-Prone MRL/*lpr* Mice

**Figure 2:**
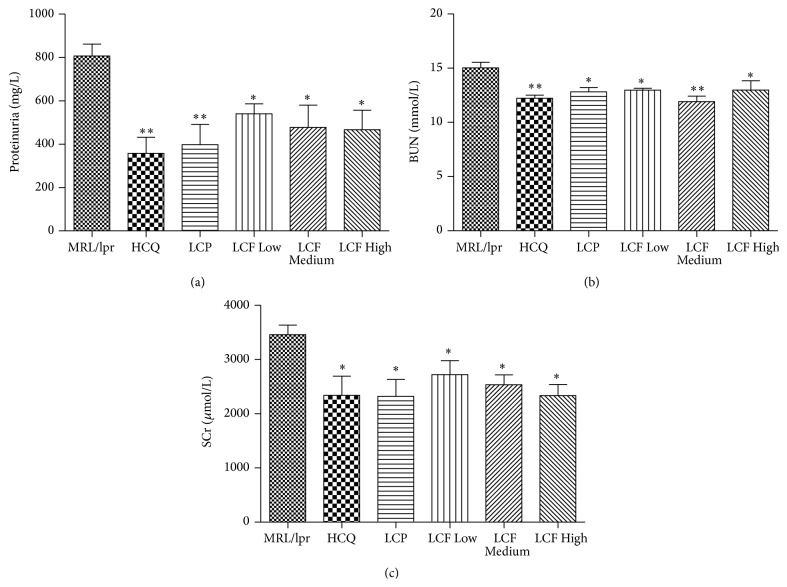
LCF granule alleviated the renal injury of the lupus-prone MRL/*lpr* mice. Urinary protein (a), BUN (b), and SCr (c) levels were determined using commercial kits to evaluate the renal function. MRL/*lpr*: lupus model group; HCQ: hydroxychloroquine sulfate tablet group; LCP: Langchuang Pill group; LCF Low: low dose of LCF granule group (0.97 g/kg/d); LCF Medium: medium dose of LCF granule group (1.95 g/kg/d); LCF High: high dose of LCF granule group (3.90 g/kg/d). Data are expressed as means ± SDs, *n* = 10. ^*∗*^ *P* < 0.05, ^*∗∗*^ *P* < 0.01 versus MRL/*lpr*.

SLE is an autoimmune disease characterized by fatal nephritis [21]. It has been reported that 50% of MRL/*lpr* mice die by 24 weeks of age, primarily because of renal failure [22]. To assess the effects of LCF granule on kidney function, proteinuria, BUN, and SCr levels were then detected. As shown in Figure 2, compared to the model group, LCF granule treatment evidently reduced the proteinuria, BUN, and SCr levels in MRL/*lpr* mice at the dosages of 0.97 g/kg/d, 1.95 g/kg/d, and 3.90 g/kg/d.

### 3.6. The Effects of LCF Granule on Protein Expression of p65 NF-*κ*B, Sirt1, and Nrf2 in the Kidney Tissues of MRL/*lpr* Mice

**Figure 3:**
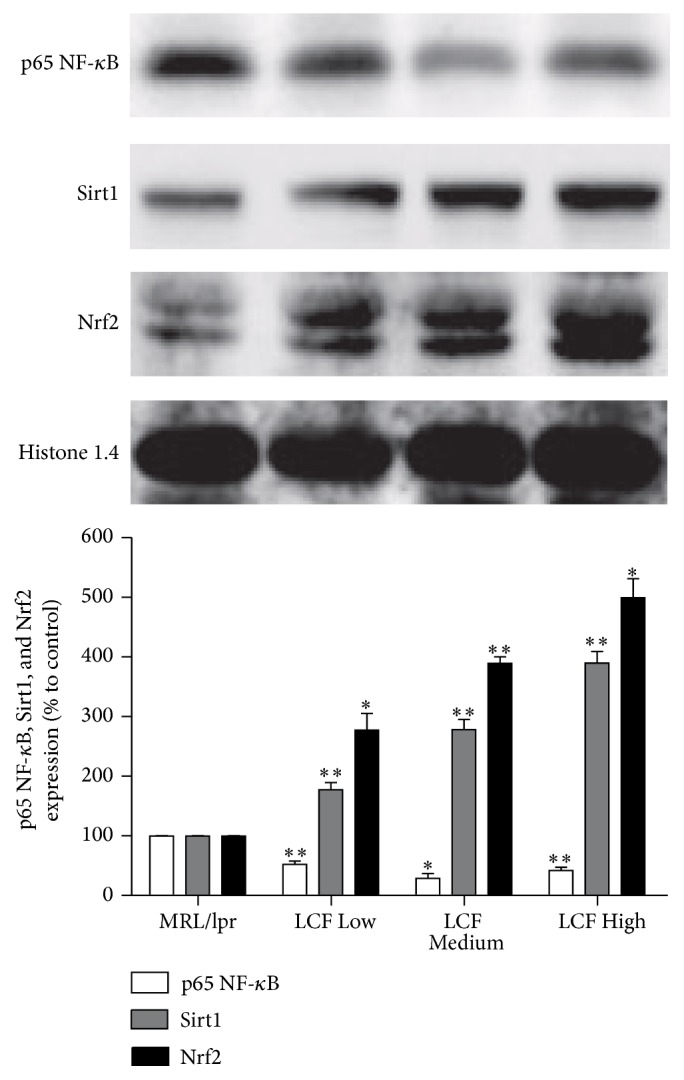
The effects of LCF granule on protein expression of p65 NF-*κ*B, Sirt1, and Nrf2 in the kidney tissues of MRL/*lpr* mice. Western blot analysis was performed to detect the protein expression of p65 NF-*κ*B, Sirt1, and Nrf2 in the kidney tissues of MRL/*lpr* mice. MRL/*lpr*: lupus model group; LCF Low: low dose of LCF granule group (0.97 g/kg/d); LCF Medium: medium dose of LCF granule group (1.95 g/kg/d); LCF High: high dose of LCF granule group (3.90 g/kg/d). Data are expressed as fold change over the control group (means ± SDs, *n* = 10). ^*∗*^ *P* < 0.05, ^*∗∗*^ *P* < 0.01 versus MRL/*lpr*.

To understand how LCF granule interferes with the renal injury process, we measured the protein levels of transcription factor nuclear factor-*κ*B (NF-*κ*B), Sirtuin 1 (Sirt1), and NF-E2-related factor 2 (Nrf2) in the renal cortex fragments of the above treated MRL/*lpr* mice. As expected, a significant lower p65 NF-*κ*B levels were found in the kidney tissues of LCF granule-treated MRL/*lpr* mice as compared to the control mice (Figure 3). Also, LCF granule treatment led to much higher levels of Sirt1 and Nrf2, two key modulators that inhibit the pathological progression of SLE and LN, in the kidney tissues of MRL/*lpr* mice (Figure 3).

## 4. Discussion

In the present study, we demonstrated the ability of LCF to alleviate the autoimmune signs of SLE in MRL/*lpr* mice. Moreover, our results also revealed that part of the beneficial effects of LCF granule on renal injury of MRL/*lpr* mice were due to modulation of NF-*κ*B signaling pathway, Sirt1, and Nrf2. This was evidenced by the downregulated p65 NF-*κ*B expression and upregulated Sirt1 and Nrf2 levels in the kidney tissues of the MRL/*lpr* mice following LCF granule treatment.

As mentioned before, the dysfunction of the immune system is crucial for SLE [6]. There are multiple immunoregulatory abnormalities, high titer of autoantibodies, systemic inflammatory response in patients with SLE, including decreased lymphocytes, maladjusted macrophages and natural killer cells, loss of immune tolerance, reduced complements, and the abnormality of lymphokines such as IL-1 [5, 6]. Female MRL/*lpr* mice are a classic animal model of SLE because they spontaneously develop autoimmune syndromes characterized by LN, hematological changes, hyperglobulinemia, marked lymphadenopathy, splenomegaly, and autoantibody formation [10, 22].

The main components of LCF are* Hedyotis diffusa *Willd.,* Arnebia euchroma *(Royle) Johnst.,* Scutellaria barbata *D. Don,* Rehmannia glutinosa *Libosch,* Salvia miltiorrhiza* Bge., and so on, which functioned in “heat-clearing and detoxifying, promoting blood circulation and removing blood stasis” [15]. Through literature reviewing, we found that kaempferol, a key ingredient in* Hedyotis diffusa* Willd., can enhance the suppressive function of Treg cells by inhibiting FOXP3 phosphorylation, which prevent the progression of inflammatory diseases, such as SLE and rheumatoid arthritis [23]. Shikonin in* Arnebia euchroma *(Royle) Johnst. exerts specific anti-inflammatory effects via inhibiting the NF-*κ*B signaling pathway and T lymphocytes activation [24–26]. Furthermore, Shikonin has therapeutic effects on LN in NZB/WF1 mice [27]. Quercetin, isolated from both* Hedyotis diffusa *Willd.* and Scutellaria barbata *D. Don, can bind to calcineurin at a region similar to cyclosporine A and tacrolimus and decrease IL-2 expression [28]. And another component of* Scutellaria barbata *D. Don, Apigenin, can suppress lupus by inhibiting autoantigen presentation for expansion of autoreactive Th1 and Th17 cells [29]. In this study, we demonstrated that the spleen and thymus coefficients were reduced by LCF granule treatment in MRL/*lpr* mice, and the CRP and anti-dsDNA autoantibody levels were also decreased. Additionally, LCF increased the CH50 level as compared with the control group. We reported for the first time that,* in vivo*, LCF modulates immune function and attenuates the activity of SLE, adding a new dimension to its immunotherapeutic potential.

LN is one of the most severe symptoms related to the cause of death in human SLE, and the prevention of developing LN is very important for improving the prognosis [21]. Our study demonstrated that the levels of proteinuria, BUN, and SCr decreased significantly in LCF granule-treated MRL/*lpr* mice. However, the reason for these effects of LCF is still uncertain. NF-*κ*B is a pleiotropic transcription factor regulating the gene expression of several adhesion molecules, cytokines, and chemotactic proteins involved in inflammation and immune response [30]. NF-*κ*B is activated in glomerular resident cells and macrophages in human renal biopsies with LN [31]. Pharmacological inhibition of NF-*κ*B in* FcγRIIb*-deficient mice can reduce the susceptibility to SLE and prevent symptoms, such as anti-nuclear antibody and kidney damage [32]. Sirt1 is a protein deacetylase that plays crucial role in inflammation and autoimmune diseases [33, 34]. The* Sirt1*-null mice develop an autoimmune-like condition, which resembles SLE [33, 34]. The livers and kidneys of* Sirt1*-null mice accumulate immune complexes, and many animals develop anti-nuclear antibodies [33, 34]. Moreover, Sirt1 exerted powerful renoprotective effects by inhibiting renal fibrosis, reducing cell proliferation, and decreasing urine protein [35]. The transcription factor Nrf2 is a major regulator of the antioxidant response and is a primary cellular defense mechanism [36].* Nrf2*-deficiency female mice develop lupus-like autoimmune nephritis [36]. And Nrf2 activation can suppress LN through inhibition of oxidative injury and the NF-*κ*B-mediated inflammatory response [37]. Tsai believed that Nrf2 activator may be a therapeutic target for severe LN treatment [38]. Overall, it is concluded that NF-*κ*B, Sirt1, and Nrf2 are crucial mediators in the pathological development of LN. Drugs that modulate these molecular signals might be of great significance for treating LN. Here, we revealed that LCF granule downregulated p65 NF-*κ*B expression and upregulated Sirt1 and Nrf2 levels in the kidney tissues of the MRL/*lpr* mice, which elucidates the beneficial effects of LCF on renal injury.

## 5. Conclusion

Taken together, the present study has shown that LCF had therapeutic effects on MRL-mediated immune dysfunction and kidney injury. Its mechanisms may be associated with modulating immune system dysfunction and regulating key signaling molecules in kidney, which clinically provide a potential therapeutic mechanism for SLE treatment.

## Figures and Tables

**Table 1:** LCF granule treatment reduced spleen and thymus coefficients in MRL/*lpr* mice.

Groups	Spleen coefficient (g/100 g)	Thymus coefficient (g/100 g)
MRL/*lpr*	0.533 ± 0.057	0.320 ± 0.027
HCQ	0.437 ± 0.034	0.282 ± 0.014
LCP	0.395 ± 0.027^*∗*^	0.273 ± 0.019
LCF Low	0.453 ± 0.027	0.261 ± 0.025
LCF Medium	0.423 ± 0.033	0.232 ± 0.020^*∗*^
LCF High	0.387 ± 0.030^*∗*^	0.289 ± 0.016

MRL/*lpr*: lupus model group; HCQ: hydroxychloroquine sulfate tablet group; LCP: Langchuang Pill group; LCF Low: low dose of LCF granule group (0.97 g/kg/d); LCF Medium: medium dose of LCF granule group (1.95 g/kg/d); LCF High: high dose of LCF granule group (3.90 g/kg/d). Data are expressed as means ± SDs, *n* = 10. ^*∗*^ *P* < 0.05 versus MRL/*lpr*.

**Table 2:** The effects of LCF granule on CD3^+^, CD8^+^, and CD54^+^ expression on the surface of peripheral blood lymphocytes in MRL/*lpr* mice.

Groups	CD3^+^ (%)	CD8^+^ (%)	CD54^+^ (%)
MRL/*lpr*	11.231 ± 4.252	7.417 ± 3.730	26.668 ± 7.310
HCQ	15.204 ± 8.576	12.196 ± 8.631^*∗*^	16.930 ± 4.870^*∗*^
LCP	7.669 ± 3.218	4.684 ± 1.941	23.615 ± 8.555
LCF Low	6.424 ± 3.357	3.052 ± 1.874	28.787 ± 10.069
LCF Medium	8.468 ± 2.796	6.407 ± 2.158	25.519 ± 5.828
LCF High	8.320 ± 4.932	5.054 ± 3.523	26.453 ± 6.459

MRL/*lpr*: lupus model group; HCQ: hydroxychloroquine sulfate tablet group; LCP: Langchuang Pill group; LCF Low: low dose of LCF granule group (0.97 g/kg/d); LCF Medium: medium dose of LCF granule group (1.95 g/kg/d); LCF High: high dose of LCF granule group (3.90 g/kg/d). Data are expressed as means ± SDs, *n* = 10. ^*∗*^ *P* < 0.05 versus MRL/*lpr*.

**Table 3:** The effects of LCF granule on anti-dsDNA autoantibody production and CH50 levels.

Groups	anti-dsDNA autoantibody (IU/mL)	CH50 (U/L)	IgE (ng/mL)
MRL/*lpr*	1216.610 ± 584.472	13.406 ± 3.776	39.269 ± 18.005
HCQ	484.913 ± 115.394^*∗∗*^	16.700 ± 4.582^*∗∗*^	33.284 ± 16.444
LCP	719.336 ± 229.275^*∗∗*^	17.484 ± 3.965^*∗∗*^	37.815 ± 10.451
LCF Low	809.469 ± 211.999^*∗*^	17.212 ± 4.339^*∗∗*^	35.518 ± 17.325
LCF Medium	879.090 ± 366.697^*∗*^	17.040 ± 4.107^*∗∗*^	34.761 ± 17.893
LCF High	696.713 ± 127.242^*∗∗*^	16.971 ± 3.905^*∗∗*^	32.982 ± 15.000

MRL/*lpr*: lupus model group; HCQ: hydroxychloroquine sulfate tablet group; LCP: Langchuang Pill group; LCF Low: low dose of LCF granule group (0.97 g/kg/d); LCF Medium: medium dose of LCF granule group (1.95 g/kg/d); LCF High: high dose of LCF granule group (3.90 g/kg/d). Data are expressed as means ± SDs, *n* = 10. ^*∗*^ *P* < 0.05, ^*∗∗*^ *P* < 0.01 versus MRL/*lpr*.
